# Intelligent Digital Twins for Personalized Migraine Care

**DOI:** 10.3390/jpm13081255

**Published:** 2023-08-13

**Authors:** Parisa Gazerani

**Affiliations:** 1 Department of Life Sciences and Health, Faculty of Health Sciences, Oslo Metropolitan University, 0130 Oslo, Norway; parisaga@oslomet.no; 2 Centre for Intelligent Musculoskeletal Health (CIM), Faculty of Health Sciences, Oslo Metropolitan University, 0130 Oslo, Norway; 3 Department of Health Science and Technology, Faculty of Medicine, Aalborg University, 9260 Gistrup, Denmark

**Keywords:** intelligent digital twins, digital twins, migraine, artificial intelligence, machine learning, deep learning, the Internet of Things, cloud computing, patient-centric, personalized

## Abstract

Intelligent digital twins closely resemble their real-life counterparts. In health and medical care, they enable the real-time monitoring of patients, whereby large amounts of data can be collected to produce actionable information. These powerful tools are constructed with the aid of artificial intelligence, machine learning, and deep learning; the Internet of Things; and cloud computing to collect a diverse range of digital data (e.g., from digital patient journals, wearable sensors, and digitized monitoring equipment or processes), which can provide information on the health conditions and therapeutic responses of their physical twins. Intelligent digital twins can enable data-driven clinical decision making and advance the realization of personalized care. Migraines are a highly prevalent and complex neurological disorder affecting people of all ages, genders, and geographical locations. It is ranked among the top disabling diseases, with substantial negative personal and societal impacts, but the current treatment strategies are suboptimal. Personalized care for migraines has been suggested to optimize their treatment. The implementation of intelligent digital twins for migraine care can theoretically be beneficial in supporting patient-centric care management. It is also expected that the implementation of intelligent digital twins will reduce costs in the long run and enhance treatment effectiveness. This study briefly reviews the concept of digital twins and the available literature on digital twins for health disorders such as neurological diseases. Based on these, the potential construction and utility of digital twins for migraines will then be presented. The potential and challenges when implementing intelligent digital twins for the future management of migraines are also discussed.

## 1. The Digital Twin Concept

The digital twin concept describes an identical virtual counterpart of a physical entity, with operational data connections between the two [1,2]. Ideally, a virtual counterpart replicates the behavior of the physical entity, thereby enabling the timely resolution of potential errors. Originally, the concept of a digital twin was applied to predict and solve engineering and manufacturing problems [3]. Michael Grieves [4,5], the father of digital twins, introduced this concept to the management of a product life cycle. According to a systematic review [6], digital twins consist of three main elements: (1) a real space where a physical entity is present, for example, an object or a person; (2) a virtual space where a virtual counterpart of the physical entity is modeled; and (3) a data connector between the two spaces to allow a bidirectional connection [6]. Recently, intelligent digital twins have been introduced that can enhance the capability of traditional digital twins and advance the management of complex systems [7]. Intelligent digital twins can provide active and continuous insights to augment human decision-making processes. This ability can improve efficiency, minimize the use of physical resources, and, ultimately, lead to better data-driven decision making and overall outcomes [4]. A front-running simulation (FRS), which is based on faster simulations than real-time occurrences to predict events, is an example of intelligent digital twins [7]. Therefore, it can help explore a wide range of scenarios, calculate probabilities, and provide estimates of outcomes [8]. It has been suggested that the installation of a digital twin framework consists of integrating real-time and conceptualized elements, running intelligent simulations and analytics, and performing interactive decisions and visualization [7,8]. 

Digital twins have attracted research and business attention in a wide range of engineering and manufacturing fields [3,9,10]. A systematic review [11] presented the characteristics of digital twins, their application potentials, the current gaps in our knowledge, and directions for future research. Interestingly, digital twins have become one of the most discussed technological applications within the domain of digital health [12]. A rapid literature review [13] provided insights into the status of digital twins within healthcare.

## 2. The Digital Twin Concept within Healthcare

The concept of digital twins has gained traction in the healthcare domain [14,15,16], in which digital human twins that resemble virtual copies of patients are created to transform patient care [17]. In the healthcare domain, digital twins are also referred to as health digital twins (HDTs) [18,19]. Lupton raised several critical considerations about the use of digital twins in health and medicine [20], which were in line with the valuable points asserted by Braun [21]. Both scholars [20,21] are well respected, and the suggested alternative terms by Lupton [20], i.e., simulation or computerized models instead of digital twins, can be considered. In this paper, the term digital twins is used to be consistent with the predominant term used in the existing literature and to avoid confusion. 

It has been proposed that digital twins can have applications in personalized medicine and precision medicine [17,19], drug development [22], precision nutrition [23], and health innovation [14,24,25,26]. The full potential of digital twins in the healthcare domain is, however, not yet realized, which might be—at least in part—due to technical, regulatory, and ethical hurdles [27,28,29]. It is expected that, by overcoming these challenges [30], digital twins will be widely embraced to facilitate connected care and alter healthcare management in the future. The Internet of Things, artificial intelligence, machine learning, deep learning, and cloud computing are among the essential technologies for designing fully functional intelligent digital twins [31,32,33,34,35,36]. According to IBM, “*the Internet of Things is a network of physical devices, vehicles, appliances, and other physical objects that are embedded with sensors, software, and network connectivity that allows them to collect and share data*”. Kelly et al. [31] showed that the Internet of Things can potentially improve health service delivery and global healthcare in the next decade. The collection of big data, especially for chronic disorders, and securing a storage system in the cloud computing environment are also proposed to reduce storage costs and provide powerful computing and quick audits while potentially increasing storage security. Therefore, they seem essential for the concept of digital twins for healthcare purposes. 

Two aspects have been proposed for digital twins in the health and medical domain: (1) digital twins as a data generator (e.g., the simulation of patients’ conditions and therapeutic responses and multiscale modeling from cells to the whole body [37]) and (2) digital twins as a data source in combination with other data sources (e.g., retrospective data and patient cohort data) for further data processing via artificial intelligence and machine learning [38]. Within this framework, various intervention types can be simulated, where different internal and external conditions can be modeled, for example, environmental changes, hormonal variations, dietary interventions, surgical operations, and therapeutic interventions, such as gene therapy and pharmacotherapy [39]. Such simulations will potentially allow the most accurate and dedicated predictions for targeted patient groups [30,39]. Wearable sensors that can provide records of digital tracking have been seen for some time now and are popular in healthcare [40,41]. The incorporation of collected data from these resources is among the initial framework of digital twins in healthcare. Digital twins of organs and tissues have also been introduced [42]. Some studies have named such digital twins as biological digital twins to emphasize the complexity of biological systems and highlight the focus of this care [39]. 

The potential applications of digital twins in healthcare are presented in Table 1. The domains in the table are defined according to the six domains of healthcare quality defined by the Institute of Medicine (IOM) [43], i.e., safety, effectiveness, patient-centered care, timeliness, equity, and efficiency.

## 3. Examples of Digital Twin Applications within Healthcare

The literature on digital twin applications in healthcare is, nevertheless, new [12]. It has been reported that digital twins can be used in multiple areas of healthcare, including personalized medicine [17], precision medicine, clinical trial design, hospital operations [25], and medical education [44], in addition to medical devices or pharmaceutical development [12]. 

Within personalized medicine [17], the great potential of digital twins has been presented for the treatment of cancer [45,46], stroke [47], multiple sclerosis [48], and cardiovascular diseases [49], to mention a few examples. The integration of digital twins in healthcare is proposed as being able to maximize the efficiency of the care by shifting from the current standard practice to individual-centered therapy [49,50]. In the case of cancer care within the oncology domain [45,51], including pediatric cancer care [52], it is proposed that virtual entities of cancer patients can be used in predictive analytics, consolidating clinical options, cancer care modeling, precision medicine, and cancer care research [52]. Digital twins for neurological disorders have also been described. For example, a proof-of-concept study presented a digital twin for stroke patients that utilized electroencephalogram (EEG) data and machine learning models [47] to act as a clinical support system for stroke prevention and post-stroke care. 

From personalized care [17] to precision public health [53] and the application of precision medicine in larger populations [19], digital twins have also been proposed as beneficial for use in various medical domains, such as viral infection, trauma, diabetes, and brain disorders [14]. Collectively, the available evidence shows that digital twins can be used in healthcare [12,53]. The ultimate goal is to realize true personalized and precision medicine [17,54]. Technical, ethical, social, and financial limitations [29], however, have slowed down the process of constructing and using digital twins in healthcare systems.

## 4. Digital Twin Concept for Personalized Care for Migraines

As mentioned earlier, the use of digital twins has been considered for neurological disorders, including multiple sclerosis [48], Alzheimer’s disease, dementia [55], and stroke [47,56], as well as for advancing personalized care. Currently, no digital twin exists for migraines; however, in theory, digital twins can be constructed and utilized for migraine care [57]. Personalized care for migraines [58] and a patient-centric approach to migraine management [59] have indeed been suggested. Therefore, digital twins can also be rationalized as being able to foster personalized medicine for migraines. 

Migraines are a highly prevalent and complex neurological disorder that appear with a variety of symptoms, including headaches and sensory disturbances [60]. Migraines affect millions of people around the world [61] and are ranked among the top disabling diseases [62], with negative personal and societal impacts [63]. The current treatment strategies are suboptimal, and no single therapeutic strategy is effective. Previous studies have attempted to identify the pathophysiological aspects of migraines to provide effective management through mechanism-based treatment [64,65]. Interestingly, migraine symptoms may change over a patient’s course of life, with fluctuations reported from one attack of a migraine to another within the same patient [66,67]. Therefore, genetics [68] alone cannot explain the pathogenesis of migraines. Epigenetics [69,70] and lifestyle factors [71], such as dietary factors [72,73], have been reported to be determinant factors in migraines. 

Biomarkers of migraines [74,75] have long been a focus of research for understanding the pathogenesis of migraines and their management. For example, bio-based biomarkers, such as miRNAs, have been investigated in this regard. A recent systematic review [76] summarized microRNAs that have been studied for the diagnosis and therapy of migraines. The dysregulation of multiple miRNAs has been identified, and miR-34a, miR-382, and miR-155 have been reported as promising biomarkers. It is important to mention that alterations in biomarkers are indicative of various features of migraines, including the type of migraine, phase of the migraine, and response to treatments. Biomarkers of migraines are not limited to biological biomarkers and genetics, and epigenetic and neuroimaging biomarkers have been investigated [74]. Recently, a registry for migraines was initiated to collect data and discover migraine biomarkers [77]. This registry, named REFORM (Registry for Migraine Study), was established to find biomarkers that are predictive of responses to erenumab [78]. The registered information includes clinical data, biological biomarkers, data from structural and functional brain images, and data on responses to a provocation test induced by calcitonin gene-related peptide [77]. Biomarkers are indicators that are measured objectively to identify physiological or pathological processes, as well as responses to pharmacological interventions [79]. 

The digital transformation and availability of smartphones and wearable devices have advanced digital healthcare [80], including digital care for migraines. For example, Ingvaldsen et al. [81] investigated an mHealth app for biofeedback treatment for adults with migraines. In line with digital care, digital biomarkers [82] are biomarkers that are measured and collected by digital devices, for example, wearable or implantable devices. Digital biomarkers are proposed to be essential elements for deep digital phenotyping [83]. Various wearable devices are available for collecting digital information, such as biosignals (e.g., heart rate and blood pressure), sleep patterns, weight, and mental health status. Research is ongoing on wearable healthcare devices and how machine learning and other technologies can use data from wearables [84].

Because digital technologies are becoming more affordable and accessible to a larger population, and the fact that some data can automatically be collected, e.g., by wearables, the collection of large amounts of data in a longitudinal manner has become possible. In addition, patient-reported measures can be collected via a digital infrastructure and combined with other digital measures to create a digital multidimensional dataset. Combining real-world digital data with clinical data, laboratory data, and omics data can create a rich digital depository for the construction of digital twins. Interestingly, the term “digitosome” has been used in the literature to describe collective digital data from an individual, which can objectively provide insights about that individual and unfold the full potential of personalized care [83].

For the construction of digital twins for migraines, a stepwise development effort is being seen. This means, for example, that some researchers have used wearable devices to collect physiological data (such as heart rate, skin temperature, muscle tension, and blood pressure) that can help in the prediction or avoidance of headaches. In a preliminary study, the Empatica 24/7 device was used to collect long-term and real-time data for migraine detection [85]. In this study, the Empatica E4 device was used to collect so-called biosignals, such as blood volume pulse, optical heart rate, temperature, acceleration, and electrodermal activity [85]. The authors aimed to construct adaptive models with the aid of machine learning, whereby versatile analysis models can help individuals affected by migraines to understand their own symptoms. In addition, migraine attacks can be predicted with the use of these models. Such models use a combination of data, such as SpO_2_, skin temperature, heart rate, and electrodermal activity, collected via wearable sensors [85]. Wearable data can also be in the form of EEG data. Cao et al. [86] used a wearable headband EEG device. They found that occipital EEG entropy was higher in migraine patients in the interictal phase and lower when they were in the preictal phase. Moreover, the transitional variation in EEG was lower in the preictal phase compared to the interictal phase. These researchers [86] concluded that the developed inherent fuzzy entropy model might prove beneficial in the future, providing a preictal alert to migraine patients and eventually leading to the detection of migraine attacks. 

The current research shows that artificial intelligence, simulation techniques, augmented and virtual reality, the Internet of Things, and cloud computing are becoming more popular in migraine research to facilitate the discovery of novel management options. For example, Zhu and Dave [87] used various machine learning models to predict migraine occurrences. They applied a data augmentation technique to a publicly available database and identified patterns and the most significant factors for predicting the occurrence of a migraine attack [87]. These researchers [87] anticipated that federated learning can enable the use of their technique toward building individualized machine learning models based on individual migraine triggers. Achieving this goal, however, needs a larger dataset to make such models more accurate. Mohan and Mukherjee [88] also pointed to the usefulness of applications of the Internet of Things and machine learning-based predictive models for predicting migraine attacks with a high accuracy. They [88] proposed that personal smart devices with the application of the Internet of Things can help in collecting individualized data, such as environmental triggers of migraines, in real time for an accurate prediction of migraine attacks. MigraineCloud, proposed by this group of researchers, uses an advanced detection app for connecting personal Internet of Things smart devices [88]. This app can predict the onset of a migraine for a particular patient by using deep learning neural networks [88]. Stubberud et al. [89] presented a successful predictive modeling in a recent prospective study with 18 patients with migraines. To model and forecast headaches, these researchers combined mobile app headache diary information and wearable readouts of their heart rate, peripheral skin temperature, and muscle tension, with mobile-based biofeedback data. Based on their findings, they proposed that high-dimensional modeling using machine learning and mobile health data could predict headaches with higher accuracy [89]. Interestingly, high-dimensional modeling has also been proposed for data-driven machine prescriptions for chronic migraines [90]. 

Collectively, the abovementioned examples show that the collection and analysis of multidimensional digital data can help resolve the problems resulting from the unpredictable nature of migraines or offer a better choice of drug. Unpredictability is a complicated challenge, particularly for complex disorders such as migraines, where many factors are involved and such factors vary among individuals. In addition, the disease features show variations and fluctuations over time depending on age, sex, and other comorbidities. Moreover, the treatment options are not optimal, and non-responders complicate the treatment process for migraines. Considering these unique features of migraines as a multifaceted disorder, personalized care seems an optimal solution. However, the development and application of personalized care for migraines has not been successful so far, and digital twins might help achieve this goal. This is proposed because digital twins can accommodate various types of data, allowing for powerful predictive models that show accurate results. To collect, store, and analyze large amounts of multidimensional data, new digital technologies will be employed in the construction of digital twins for migraines. For instance, it is expected that cloud computing will be used in constructing digital twins for migraine treatment [91]. This will serve as a powerful platform for digital twins in terms of servers for computing and storing large amounts of data obtained via various digital tools (e.g., wearables). Moreover, fast data processing and the capability of presenting analytical results are further advantages of cloud computing. The Internet of Things [92] can enable communication via network transmissions of data [93], which are essential for efficient digital connections and real-time data transfer [94]. These technologies would enable the realization of the concept of intelligent digital twins [7]. An intelligent digital twin is a data-driven entity [95]; therefore, data structures and the incorporation of big data analyses [96] seem crucial for predictive tasks and the determination of interventions that can achieve optimal responses [15]. For digital twins, modeling and simulations [97] are essential to fully map their physical counterparts, because these processes can add data on real phenomena occurring in the physical counterparts of the virtual twins [98]. In the context of digital twins, coupling with artificial intelligence [99] will be critical for improving their performance [100]. Innovative and informative visualization systems have also been integrated with digital twins to enhance their performance, for example, virtual reality, augmented reality, and mixed reality. Extended reality has, in fact, been investigated for relaxation training combined with wearable neurofeedback in children with migraines [101]. A similar attempt with the use of a portable biofeedback virtual reality device resulted in decreases in analgesic use and lower depression in adult patients with chronic migraines [102].

## 5. Other Applications of Digital Twins for Migraines

In addition to the huge advantage of achieving personalized care for migraines, various other benefits can be expected with the aid of intelligent digital twins. Theoretically, digital twins can be implemented for a better understanding of migraines [74]. The multidimensional and dynamic features of migraines, which are highly influenced by various internal and external factors, show a complicated picture regarding understanding migraines as a disorder. Therefore, digital twins can help to understand these features by investigating thousands of scenarios in replicas and analyzing data. For example, the use of intelligent digital twins can help obtain a better understanding of the triggers, lifestyle factors, drug–drug interactions, and comorbidities in a patient’s journey [64]. Lifestyle modifications to prevent migraines are also practically challenging in a physical space; however, in a digital space, factors, including diet, sleep, and exercise, can be tested more rapidly in various scenarios to identify the optimal solutions. The determination of an optimal response to medication and dose adjustments for a better response or safety outcomes can also be empowered by the aid of digital twins. Ideally, genetics and environmental factors that contribute to migraine pathogenesis and responses to various treatment strategies can be investigated and understood. Managing the coappearance of other diseases with migraines and the need for consuming several medications at the same time can be highly challenging. The application of digital twins can provide benefits from diagnosis to the treatment of migraines [57], because such an application makes it possible to examine different treatment plans with digital twins and predict prognosis and medication responses.

Interestingly, artificial intelligence has been used for the classification of headaches and migraine stages [86,103,104]. For example, one study [105] applied machine learning to analyze patient-reported symptoms to investigate if the classification of headache disorders can be automated. Data from over 2000 patients were merged into five entities, and a stacked classifier model was applied with four levels [105]. In the model, the first layer was between migraines and other headaches, the second layer was between tension-type headaches and other headaches, the third layer was between trigeminal autonomic cephalalgia and other headaches, and the fourth was between epicranial and thunderclap headaches [105]. The stacked classifier in this study [105] showed an accuracy of 81%, a sensitivity of 88%, and a specificity of 95% for migraines. Therefore, it seems feasible to utilize machine learning in headache classification based on analyzing patient-reported data [105]. Other studies have tried to use artificial intelligence and machine learning for the subtype classification of migraines, e.g., migraines with aura. For instance, a study [106] tested different machine learning approaches to differentiate healthy patients from those with migraines with aura. This study [106] employed magnetic resonance imaging data, including cortical thickness and volume, and the algorithm resulted in 97% accuracy in the migraine with aura classification and 98% accuracy for the distinction between simple and complex aura. 

Patients with migraines may overuse analgesics and present medication overuse headaches. Medication overuse headaches [107] are classified as secondary headaches as a consequence of headache medications overuse by an individual who has a primary headache disorder, such as migraines or tension-type headaches. Artificial intelligence and machine learning techniques have been able to predict medication overuse in migraine patients [108]. Ferroni et al. [108] employed a decision support system built upon a machine learning model to extract prognostic information from demographic, clinical, and biochemical data and predicted medication overuse in migraine patients with a high accuracy (87%).

The identification of the maximum and minimum pain levels in migraines and rs-fMRI (resting-state functional magnetic resonance imaging) classification have also been performed with the aid of artificial intelligence and deep learning [109,110]. Migraine prevention has been investigated with the aid of linear discriminant analysis (LDA), along with the application of the Internet of Things and machine learning [111]. LDA is a method that usually is used when datasets have multidimensions or appear with a large number of features, for example, brain imaging data [112]. Trigger detection and prediction of the onset of a migraine have been studied with the aid of deep learning and cloud computing by applying artificial intelligence and the Internet of Things [88]. In a previous study, a virtual reality technique was applied to study migraines and how their symptoms affect patients via visual simulation in a virtual environment [113]. Migraine symptoms have also been mimicked in high- and low-immersion conditions via the application of augmented reality [114]. 

These examples collectively point to several isolated existing solutions for advancing the understanding of migraines and the provision of better migraine care. The current challenge is, however, to unify the existing solutions into one combined solution. Considering the potential and promising reports of applying digital twins in other disorders, such as cancer [45], cardiovascular disorders [49,115], and neurological disorders [47,48,55], it is likely that digital twins can become integrated into migraine care. 

Digital twins can also be used for drug and medical device developments. Constructing a large number of digital twins can help clinical trial designs by allowing virtual tests of drug effects and side effects. Potentially, this strategy can help in determining drug dosages in humans with acceptable accuracy. The challenges of drug–drug and drug–diet interactions or medication overuse also seem to be handled more appropriately if a digital twin of a patient can be constructed. Perhaps the management of migraines and the application of medical devices in treatment strategies can also be tested with the aid of digital twins, similar to what is hypothesized for drug treatments. 

Digital twins can also advance migraine research. A similar approach has been used for cardiovascular disorders [49]. Virtual organs, such as a virtual brain for migraines, can offer a wide range of benefits, for example, an investigation of unified brain imaging and recording methods [103], which are used for disease detection and mechanism-based treatments. 

It is also envisioned that health services can benefit from digital twin utilization in the treatment of migraines. Liu et al. [40] asserted that integrating digital twins in elderly healthcare could provide more accurate and fast services for older adults. The researchers proposed a framework for a cloud healthcare system based on digital twin healthcare (CloudDTH) and implemented it in a case study testing its feasibility for older adult patients [40]. Such a system can be customized to provide appropriate care for migraine patients.

Migraines are a highly complex [60] and somewhat unpredictable disorder. The uncertainty of their recurrent nature and the burden of the interictal phase of migraines [116] provide an opportunity for stepping outside the traditional treatment framework and enabling more advanced care. Constructing and utilizing digital twins for migraine treatment can be a way forward in this direction [57]. It is perhaps just a matter of time before the application of digital twins for migraines to optimize prevention and treatment is realized. 

**Figure 1:**
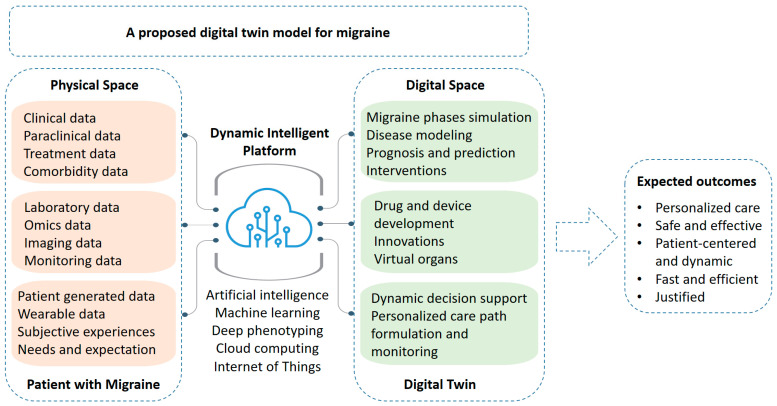
A proposed overview of the application of intelligent digital twins for the personalized care of migraines. Within the concept of a digital healthcare system with the application of digital twins, a physical space and a digital space exist. For migraines, the physical space is proposed as the space for data collection. In this space, screening, consultation, diagnosis, and treatment take place. Patient data, e.g., omics data, wearable data, and imaging data, are also often collected. This is also a place for patient education, monitoring and follow-up, medication adjustments, and offering alternative methods or medications for the optimization of management. In the digital space, with the aid of digital data from the physical space, simulation, modeling, prognosis, and various predictions can take place. Integrated data modeling, basic research (e.g., virtual organs), and virtual drug and device testing can also become feasible. Dynamic decision trees for health support and the realization of a personalized care path can therefore be utilized. Connectivity between the twins is made with the aid of advanced digital technology using a dynamic intelligent platform with acceptable security that can employ artificial intelligence, machine learning, deep learning, and cloud computing for connections, interactions, analysis, and the storage of digital data. This valuable construct is proposed to yield potential useful outcomes for the management of migraines in terms of true patient-centered personalized care that is fast and efficient with minimal errors.

A proposed overview of the application of intelligent digital twins for the personalized care of migraines is shown in Figure 1. Please note that this conceptual presentation is based on the author’s review and opinion and has not yet been implemented in an experimental or clinical setting.

## 6. Challenges of Utilizing Digital Twins in Migraine Care and Future Perspectives

Despite the many potential benefits of digital twins [19,117] outlined above for migraines, there are some challenges that must be overcome to transition from the existing solutions to digital twin-based solutions. An initial challenge is that migraine patients’ data are not collected in a qualified manner. At present, no standard is available for structuring data and data flow to facilitate the development of digital twins for migraine care. This does not necessarily mean that a new set of standards must be constructed first. Perhaps the utilization of existing standards applied for other disorders (e.g., cancer and multiple sclerosis) can be reviewed and adopted for migraines. However, it must be noted that some additional technical solutions might be required, for example, the employment of natural language processing technologies for the extraction of clinical data in a usable format from patients’ records. Security has been, and will continue to be, a general concern within digital healthcare. Blockchain technology for data sharing [118] has been suggested as a potential solution to ensure the security and traceability of data sharing [119]. The data collection phase itself needs a dedicated plan and the cooperation of an interdisciplinary team [120]. Digital twins need a user-friendly platform to facilitate communication among healthcare specialists and patients [14]. Technical challenges for the creation, maintenance, and management of digital twins must also be considered [7]. 

A gap between clinicians and data scientists [121] and trust issues would reduce the capacity for handling decisions suggested by algorithms and data interpretation. Perhaps taking shared responsibility would reduce skepticism in the clinical context [122]. A fear of clinicians being replaced by digital devices in clinics has also raised a negative atmosphere [49]. It is, therefore, essential to clarify that digital twins are support tools and are expected to enhance clinicians’ ability to deliver better care. It is often challenging to manage a multidisciplinary team consisting of clinicians, such as migraine specialists, neurologists, radiologists, psychologists, and nutritionists, and scientists, such as computer scientists, neuroscientists, and pharmacologists, who need to work closely together. However, a clear recognition of the core common interest of elevating the life quality of patients with migraines can be a helpful unifying factor.

To construct digital twins at the level of multiple organs, biosensors and nanosensors might become required and must be constructed first. In addition, the cognitive aspects of migraines cannot be neglected. Currently, appropriate cognitive intelligent systems to provide data for cognitive processes seem lacking. 

Security and privacy are also key issues, and the current solutions to overcome these challenges seem costly and complicated as a consequence of the huge amounts of data and the multiple types of technologies involved in creating intelligent digital twins. The infrastructure of the Internet of Things must also be developed and maintained, which is demanding in terms of costs and practicalities. Consequently, the costs and economic impact must be estimated for both short- and long-term benefits [29]. Concerns are still present about the cybersecurity of digital twins [29]; however, both the EU and global regulations can help impose new relevant legal requirements. 

A large body of research on the ethical issues associated with digital twins [29] is dedicated to addressing the challenges of balancing fairness, equality, and health [19,123]. At present, bias exists in healthcare-related datasets with respect to racial, gender, and other demographic features. Using skewed data to construct digital twins would increase the risk of health inequity [29] and eventually lead to a faulty recommendation system for decision making [124]. Even though the application of digital twins could exacerbate health inequity, one can imagine that they could also be used as opportunities to conduct replications and simulate data from minority groups with fewer resources [27]. A preliminary mapping study [125] showed that it is unrealistic to eliminate all ethical issues surrounding the use of digital twins in personalized care. The benefits and risks with regard to the socio-ethical aspects of using digital twins in healthcare have been discussed in the literature [29]. Accordingly, the value in utilizing digital twins can be improved in terms of disease prevention and treatment and reduced healthcare costs, and the risks that need to be addressed can be related to data privacy and property, health equality, and social justice in healthcare [29].

## 7. Concluding Remarks

Migraines are a complex and disabling disorder with a multidimensional nature that dynamically fluctuates in response to various factors during a patient’s journey. This feature demands an efficient monitoring system and the adjustment of treatment plans or preventive strategies based on each individual patient’s needs, i.e., personalized medicine. Despite various efforts, no optimal solution is available to enable efficient personalized care for migraines. Theoretically, digital twins can provide a solution. Currently, attempts to utilize digital twins in migraine care are scattered, and several barriers (e.g., technical, ethical, and societal) prevent the development of intelligent digital twins. Considering the progress made in implementing digital twins for neurological disorders, cardiovascular diseases, and cancers, it is possible to adapt some of the developed models and customize them for the initial steps of constructing digital twins for migraines. Initiatives at the national and international levels are recommended to gather specialists and policymakers in order to discuss various aspects of digital twins. Ultimately, if the benefits are considered to outweigh the risks, it would push the boundaries for the realization of intelligent digital twins in migraine care.

## Figures and Tables

**Table 1:** Examples of potential applications of digital twins within the IOM framework of healthcare quality [43] in healthcare systems.

Domain	Potential Applications
Safety	Digital twins allow the testing of various interventions on identical digital models of patients; hence, any risk can be predicted or detected before real-world interventions with patients. This capability offers safer procedures and interventions and minimizes potential harm.
Effectiveness	Digital twins allow an examination of the latest treatments, medical devices, and technologies to provide evidence regarding the effectiveness of a treatment choice and optimize disease management among patients. Decision trees and algorithms embedded in digital twins and advanced deep learning can help provide appropriate individualized choices and personalized care.
Patient-centered care	Digital twins are aligned with the concept of recognizing the uniqueness of each patient and providing personalized care. Individual aspects are taken into consideration to ensure personalized holistic decision making with the aid of digital twins. Patients’ own data are used for their own care, reflecting active patient involvement in treatment plans based on individual needs.
Timeliness	Digital twins, particularly intelligent digital twins, can provide timely actionable information for decision making due to their continuous monitoring capability and provision of real-time feedback or even early timing feedback. It is expected that intelligent digital twins can facilitate treatment plans and preventative care.
Equity	Digital twins are expected to influence equity in healthcare. Both their risks and benefits have been discussed in relation to health equity. Digital twins can close or widen the gap of equitable acts during the delivery of care. This domain is currently unknown.
Efficiency	Digital twins are speculated to reduce costs (a proper assessment and cost analysis are required) and enhance efficiency within healthcare systems in terms of workflow, waste, and long-term costs and consequences. A more efficient healthcare system can save resources by integrating digital twins and personalized care, thus reducing unsafe and/or inefficient care, complications, and readmissions. This domain is a dynamic feature and requires continuous review and monitoring to adjust to the needs for optimal efficiency.

## Data Availability

Not applicable.
